# The Placenta as the Main Source of Serotonin in Ontogenetic Dynamics: Inflammation-Induced Modulation of Placental Serotonin Can Be Prevented by Immunoglobulin Administration

**DOI:** 10.3390/ijms252413532

**Published:** 2024-12-18

**Authors:** Nadezhda Bondarenko, Nadezhda Lifantseva, Svetlana Voronova, Victoria Melnikova

**Affiliations:** Laboratory of Comparative Developmental Physiology, Koltzov Institute of Developmental Biology of the Russian Academy of Sciences, Moscow 119334, Russia; n.s.bondarenko@gmail.com (N.B.); n.lifantseva@gmail.com (N.L.); svetvor@mail.ru (S.V.)

**Keywords:** serotonin, placenta, fetus, prenatal development, LPS, immunoglobulins

## Abstract

Placental serotonin is recognized as a key component of feto-placental physiology and can be influenced by environmental factors such as maternal diet, drugs, stress, and immune activation. In this study, we compared the contribution of placental and fetal sources to the maintenance of serotonin levels required for normal fetal development during ontogenetic dynamics. Our results demonstrated the leading role of the placenta at almost all stages of development. We investigated the modulatory effect of inflammation on placental serotonin levels. The data obtained showed that the susceptibility to prenatal inflammation depends on its severity and varies considerably at different stages of development. According to our results, inflammation-induced modulation of placental serotonin levels can be prevented by immunoglobulin administration at both early and late stages of development. Disturbances in placental serotonin signaling during critical developmental periods may have long-lasting consequences for the health and behavior of the offspring. Therefore, the ability to prevent environmental modulation of placental serotonin, and hence negative effects on the developing fetus, is of great importance.

## 1. Introduction

The placenta, a multifunctional organ essential for fetal growth and survival during development, secretes a variety of hormones, growth factors, and cytokines [1,2]. Among these factors, serotonin has been recognized as a key component of placental physiology. While maternal serotonin does not cross the placental barrier, maternal tryptophan can be converted to serotonin in the placenta and delivered to the fetal circulation in both rodents and humans [3,4]. Studies in animal models have demonstrated the critical role of serotonin in the regulation of craniofacial, gastrointestinal, endocrinal, and cardiovascular morphogenesis, as well as critical neurodevelopmental processes, including neuronal cell proliferation, differentiation, migration, and brain wiring during fetal development [5,6,7,8,9,10,11,12,13,14,15]. Nevertheless, there are other sources of serotonin in embryonic tissues besides the placenta, including the fetal brain, enterochromaffin cells, mesenchymal cells, etc. [3,4,15]. The relative contribution of these sources of serotonin remains uncertain.

There is increasing evidence that the disturbance of serotonin signaling during critical developmental periods can have long-lasting consequences for the offspring [4,8,10,12,14]. Placental serotonin metabolism is sensitive to various environmental stimuli such as maternal stress, depression, or inflammation [2,4,15]. The available data describe the effects of environmental factors at specific developmental stages and are sometimes contradictory [2,4]. Our recent study in mice showed that different intensities of stress have opposite effects on placental serotonin levels [16]. Maternal inflammation in mid-pregnancy upregulates placental serotonin synthesis, whereas in late developmental stages, tryptophan metabolism shifts towards the kynurenine pathway [17,18]. The mentioned contradictions need to be clarified.

It is well-established that fetal brain development is highly sensitive to maternal inflammation, which is associated with an increased risk of several neurodevelopmental, psychiatric, cognitive, and behavioral disorders in the offspring [19,20,21]. Therefore, the ability to prevent environmental modulation of placental serotonin, and hence negative effects on the developing fetus, is of great importance. A therapeutic approach aimed at suppressing inflammatory processes by intravenous (i.v.) administration of human immunoglobulins (Igs) is well-known and has been successfully used in the clinical treatment of inflammatory disorders [22,23,24,25]. Experimental studies in mice have demonstrated the ability of both IgM and IgG to protect against lipopolysaccharide (LPS)-induced inflammation [26]. However, it remains unknown whether Igs could prevent LPS-induced modulation of placental serotonin levels.

In this study, we compared the impact of different sources of serotonin in the feto-placental unit on ontogenetic dynamics. Our results indicate the leading role of the placenta in almost all developmental stages. We demonstrate that the susceptibility to prenatal inflammation depends on its severity and varies considerably at different stages of development and that the inflammation-induced modulation of the placental serotonin level can be prevented by the administration of Igs at both early and late developmental stages.

## 2. Results

### 2.1. Comparative Analysis of the Serotonin Sources in the Feto-Placental Unit During Ontogenetic Dynamics

**Figure 1:**
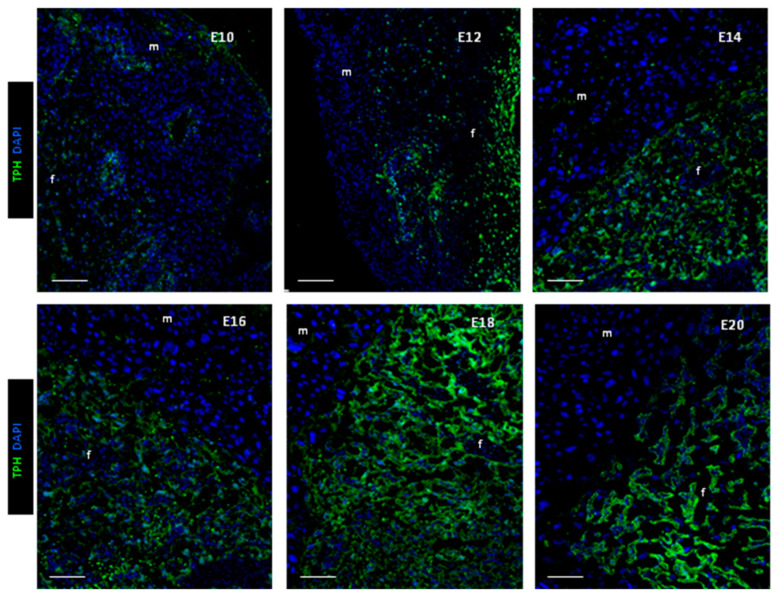
The expression of tryptophan hydroxylase (TPH) in placenta at different developmental stages revealed by immunohistochemistry. Nuclei were stained with DAPI, “f”—fetal and “m”—maternal compartments of the placenta, bar—100 μm.

The expression of the first, rate-limiting enzyme of serotonin synthesis, TPH was examined by the immunohistochemistry in placentas at stages E10–E21. According to our results, only a weak immunopositive signal was observed at stage E10 (Figure 1). TPH was observed in single cells that were scattered throughout the section as well as in association with blood vessels. By stage E12, the amount of immunopositive material increased significantly, primarily in the fetal compartment of the placenta. Therefore, our data suggest that TPH expression in the placenta is established between E10 and E12 stages. A typical distribution pattern of TPH throughout the fetal compartment of the placenta was observed from E14. TPH was observed mainly in cytotrophoblast/syncytiotrophoblast cells in the labyrinth zone and endothelial cells of the placenta (Figure 1). There was no change in the distribution pattern of TPH distribution between stages E14 and E20. Our observations were in agreement with previous studies in mice and rats [3]. Serotonin-synthesizing enzymes were detected mainly in the syncytiotrophoblastic cell layer and fetal capillaries within the labyrinth zone in both mid- and late-gestation mice and rats [3,27].

**Figure 2:**
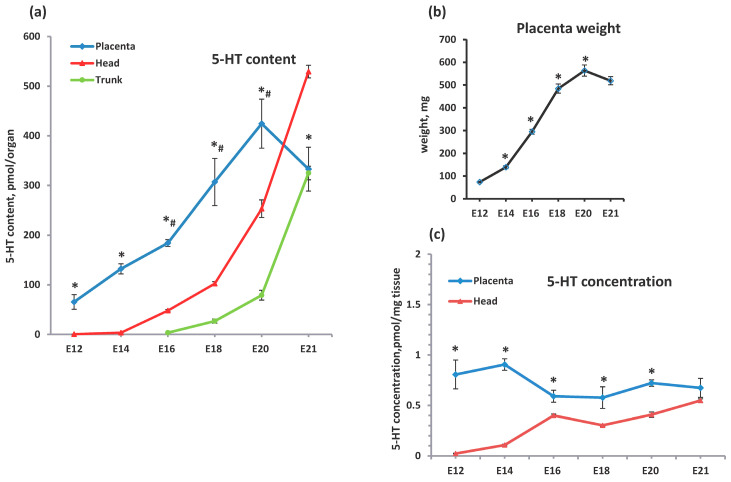
Comparative analysis of different serotonin sources in feto-placental unit during ontogenetic dynamics, (**a**) serotonin contents in the placenta and in the head and trunk of the fetus during the ontogenetic dynamics (n = 9, * *p* < 0.05 placenta vs. head, # *p* < 0.05 placenta vs. trunk at each developmental stage), (**b**) the ontogenetic dynamics of the placental weight between stages E12 and E21 (n = 12, * *p* < 0.05 vs. previous stage), (**c**), serotonin concentrations in the placenta and in the fetal head during the ontogenetic dynamics (n = 9, * *p* < 0.05 placenta vs. head at each developmental stage).

Based on our immunohistochemical data, we analyzed placental serotonin levels from E12 to E21 stages (Figure 2). The serotonin content was 65.46 ± 14.78 pmol per placenta at E12, and then progressively increased up to 424.45 ± 49.37 pmol at E20 (Figure 2a). A small, statistically insignificant decreasing trend was observed between E20 and E21 (Figure 2a).

The developmental dynamics of the placental serotonin content reflected changes in placental weight (Figure 2a,b). The weight of the placenta progressively increased from 73.5 ± 2.82 mg at E12 to 563.5 ± 24.62 mg at E20, then remained unchanged (Figure 2b). Thus, increasing placental serotonin content was mainly determined by placental growth.

Placental serotonin content significantly exceeded those in the fetal head during the period E12–E20 (Figure 2a). As serotonin in the fetal head is mainly synthesized by the brain, its levels in the head can be seen as a reflection of those found in the brain. During the E12–E14 stages, the brain’s serotonin content was extremely low (0.48 + 0.12 and 3.32 + 0.14 pm per organ, respectively) and then progressively increased up to 529.38 + 12.81 pmol at E21 (Figure 2a). The brain overtakes the placenta in serotonin levels only 1 to 1.5 days before birth (Figure 2a). The level of serotonin in the brain is consistent with the dynamics of the differentiation of serotonergic neurons. Indeed, the first serotonin-producing neurons appear in the Raffe nucleus at the E11–12 stages [7]. Their number increases throughout the prenatal period of development, reaching a maximum before birth [7]. Thus the ontogenetic dynamics of the brain’s serotonin levels reflect the maturation of the brain’s serotonergic system.

The contribution of serotonin sources from fetal peripheral tissues was significantly lower compared to the placenta and brain in developmental dynamics up to stage E20 (Figure 2a). The total serotonin content in the fetal trunk increased from 3.44 ± 0.71 at E16 to 79.01 ± 9.8 at E20 (Figure 2a). The most pronounced increase in serotonin levels in peripheral fetal tissues was observed between stages E20 and E21 (from 79.01 ± 9.8 up to 324.95 ± 13.65 pmol) (Figure 2a). Indeed, in fetuses, the main serotonin producers outside the brain are the duodenum and small intestine, but it is known that TPH expression in intestine chromaffin cells starts late in embryogenesis [28], mostly after stage E16. Thus, the contribution of serotonin from peripheral fetal tissues up to the E20 stage is negligible compared to that from the placenta and the brain.

The concentrations of serotonin both in the placenta and in the brain varied slightly during ontogenesis (Figure 2c). The placental serotonin concentration was almost the same between the E12 and E14 stages (0.8–0.9 pmol/mg tissue), then decreased up to 0.59 + 0.06 pmol/mg at E16 (Figure 2c), and remained unchanged up to the E21 stage. The serotonin concentration in the placenta exceeded that in the brain at all developmental stages except for E21 (Figure 2c). In the brain, we observed a slow increase in serotonin concentration during the developmental period studied. Similar to the placenta, the brain’s serotonin content was predominantly determined by the growth of the organ rather than by changes in serotonin concentration (Figure 2a,c). Thus, our results clearly demonstrated that the placenta is the leading serotonin source throughout most prenatal development (E12–E20).

### 2.2. LPS-Induced Modulation of Placental Serotonin Levels in Ontogenetic Dynamics

**Figure 3:**
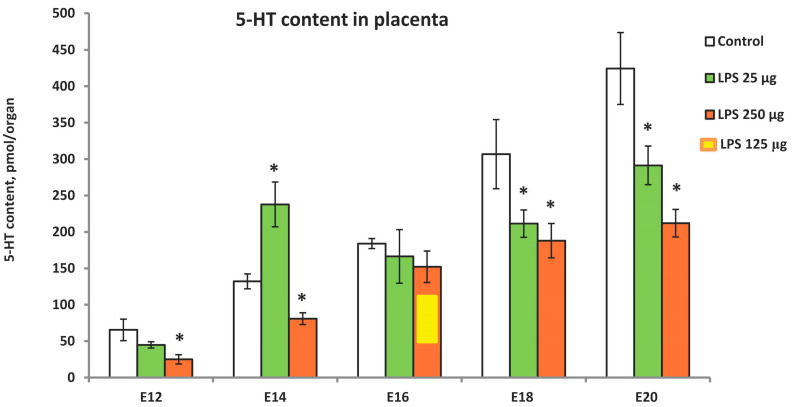
The effect of LPS administration on the placental serotonin content during ontogenetic dynamics. Pregnant rats received LPS at doses 25 or 250 μg/kg b.w., and the placental serotonin content was measured 24 h after injection. n = 8 for each experimental group, * *p* < 0.05 vs. control.

We investigated the effects of LPS administration at doses of 25 and 250 μg/kg on the placental serotonin content during the period corresponding to the leading role of the placenta as a source of serotonin (E12–E20). Placental serotonin content was measured 24 h after LPS administration (Figure 3). A high dose of LPS reduced serotonin content at all developmental stages examined except E16. At this stage, a paradoxical response to LPS was observed. As the 250 µg/kg dose resulted in high fetal lethality, a lower dose of 125 µg/kg was used, but no changes in serotonin levels were observed within 24 h (Figure 3). We cannot explain the particular vulnerability of fetuses at the E16 stage to severe inflammation, but this fact has been confirmed in three pregnant rats.

The effect of inflammation on the placental serotonin content also depended on its severity. Serotonin levels were either reduced (E18, E20) or not affected (E12, E16) by a low dose of LPS (Figure 3). An extraordinary response to a low dose of LPS was observed at the E14 developmental stage when, in contrast to other stages, placental serotonin content increased significantly. We recently observed similar effects in mice in response to stress [16]. Mild prenatal stress increased placental serotonin content at the E14 stage, whereas severe stress decreased it [16]. Our results suggest that the susceptibility of placental serotonin to environmental factors varies considerably at different stages of development, indicating the modulatory nature of the effect of inflammation on placental serotonin.

### 2.3. Immunoglobulin Administration Is Able to Prevent LPS-Induced Modulation of Placental Serotonin Levels

The potential efficacy of Igs to prevent LPS-induced modulation of placental serotonin was studied at two developmental stages—E14 and E20—when the placenta is the main source of serotonin. We applied the Igs in combination with a high dose of LPS which is known to induce a strong inflammatory response and a decrease in placental serotonin content.

**Figure 4:**
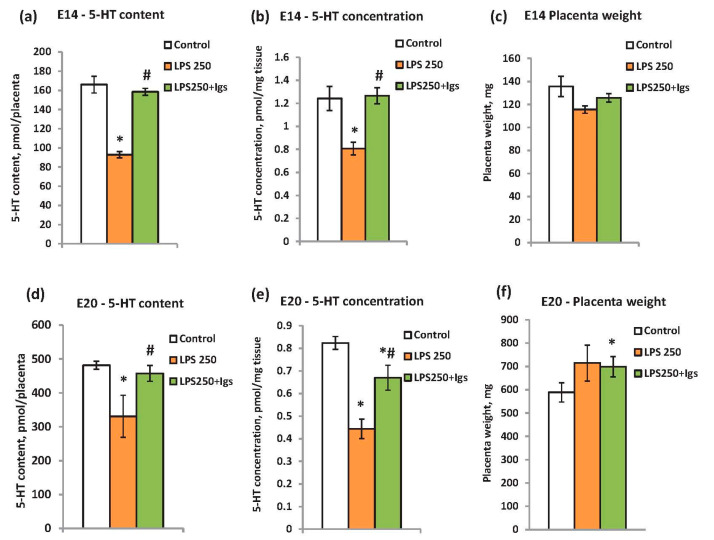
The preventive effect of immunoglobulin administration on the placental serotonin level in LPS-treated rats at E14 (**a**–**c**) and E20 (**d**–**f**). (**a**,**d**)—serotonin content per placenta, (**b**,**e**)—serotonin concentration per mg tissue, (**c**,**f**)—the weight of placenta. n = 8 for each experimental group, * *p* < 0.05 vs. control, # *p* < 0.05 between “LPS” and “LPS + Igs” groups.

According to our results, the serotonin content in LPS-treated rats was restored to the level of the control rats after Igs administration at stage E14 (Figure 4a). The same restoration was observed for the serotonin concentration per mg tissue (Figure 4b) since the weight of the placenta changed insignificantly (Figure 4c). The Igs administration to E20 rats was slightly less effective than at E14 (Figure 4d). The treatment restored the serotonin content per organ to that of the control rats (Figure 4d). However, the serotonin concentration was only partially recovered (Figure 4e). This can be explained by the increase in placenta weight in the experimental group at stage E20 (Figure 4f). It might be suggested that a higher dose of Igs should have been used at the E20 stage. Altogether, the results demonstrated that LPS-induced modulation of the placental serotonin lever can be prevented by the Igs administration.

## 3. Discussion

In this study, we demonstrated that the placenta is the main source of serotonin throughout most of prenatal development. The effect of inflammation on the placental serotonin level depends on its severity and on the developmental stage. The modulatory influence of the inflammation on placental serotonin can be prevented by the administration of Igs.

The question of the relative importance of different sources of serotonin has remained unresolved despite the intense research into placental serotonin and its role [3,13,27,29,30]. Our results clearly identified the leading role of the placenta, compared to fetal tissue, as the main source of serotonin required for proper fetal development.

In the present work, we have characterized the full age-related dynamics of both the placental serotonin content and the effect of inflammation on its levels for the first time. The ability of environmental factors to affect placental serotonin levels has been repeatedly demonstrated [17,18,31], but the available studies describe only one or two developmental stages.

The ability of serotonin to control the development of various fetal systems and its sensitivity to environmental factors allow us to consider placental serotonin as a link between the environment and the developing organism, capable of fine-tuning the fetus to changing environmental conditions. During the critical period of development, adverse environmental exposure, such as stress, toxins, and inflammation, may affect the growth trajectory before birth, significantly impact health, and increase disease susceptibility in offspring [32]. Our results demonstrated that susceptibility to environmental factors varies considerably at different stages of development. In our study, we found that at the E16 stage, rat fetuses are particularly sensitive to maternal inflammation. This was the only stage at which high fetal mortality was recorded. At this stage, however, placental serotonin levels were not affected by maternal inflammation.

According to our results, severe inflammation reduced the level of serotonin in the placenta at most of the developmental stages studied. It can be speculated that this inhibitory effect may be due to the modulation of the tryptophan metabolism pathway. In the placenta, tryptophan can be metabolized via the serotonin and kynurenine pathways. Tryptophan homeostasis is crucial for proper placental function and fetal development. It is known that placental tryptophan metabolism may be altered by maternal inflammation, stress, gestational diabetes, or preterm delivery [4,18]. Maternal inflammation as well as human intrauterine bacterial infection may switch placental tryptophan metabolism from serotonin to the kynurenine pathway and increase the placental kynurenine/tryptophan ratio [33].

It should also be noted that stage E14 was very unusual in terms of response to inflammation. In contrast to other stages studied, mild inflammation at E14 was associated with an increase in serotonin levels in the placenta. In our recent study [15], we observed a similar peculiarity of stages E13–14 in mice, where mild prenatal stress increased placental serotonin levels. As a possible explanation for this phenomenon, some recent observations may be considered. Studies in mice have demonstrated that mild maternal inflammation induced by the injection of low-dose poly(I:C) caused a transient increase in placental tryptophan level and stimulated Tph1 gene expression and THP enzymatic activity in the placenta at the E13 stage [17]. These changes led to an increase in placental and fetal serotonin contents and offspring behavioral outcomes [17]. Moreover, mild inflammation induces an elevation of proinflammatory cytokines and chemokines in the placenta, and the ability of inflammatory signals to alter neurodevelopment is well-documented [17,31,34,35]. There is evidence suggesting that the effects of mild inflammation may be mediated in part by cytokine interactions with placental toll-like receptors [17,36]. A recent study in mice demonstrates that prenatal stress leads to placental inflammation, including an elevation in the chemokine CCL2, pro-inflammatory cytokine IL6, and serotonin and tryptophan levels in the placenta [31]. Using CCL2−/− genetic knock-out (KO) mice, the authors established that placental tryptophan and serotonin availability as well as TPH activity are dependent upon CCL2 [31]. Nevertheless, the mechanisms linking maternal inflammation during pregnancy with increased placental serotonin levels and a risk of developmental abnormalities in the offspring are not yet fully understood.

The transient disruption of essential serotonin-mediated signaling events during critical developmental periods may have long-lasting developmental and physiological consequences. Therefore, the search for approaches to prevent placental serotonin fluctuations is of great importance. Our results have demonstrated the efficacy of Igs in preventing the inflammation-induced modulation of placental serotonin because of its beneficial actions on inflammatory pathologic conditions and its ability to promote tolerance to LPS [22,23,24,25,37]. It is well established that intravenous administration of Igs has a positive effect in various bacterial and viral infections [25,37]. A preparation of highly purified polyclonal and polyspecific IgG or IgM isolated from the plasma of thousands of healthy donors is currently used in a large and growing number of systemic inflammatory disorders [22,25,38]. Moreover, improved pregnancy outcomes after Igs therapy were described [32]. In experimental rodent models with LPS-induced inflammation, IgG and IgM suppressed the synthesis of proinflammatory cytokines [26]. Intrauterine inflammation is observed in about 20% of all pregnancies and has been associated with premature births and neurodevelopmental disorders [39]. Thus, our findings raise the prospect of preventing inflammation-induced modulation of placental serotonin, thereby limiting adverse effects on the offspring.

## 4. Materials and Methods

### 4.1. Animals and Experimental Design

Wistar rats (Stolbovaya Breeding Center, Moscow, Russia) weighing 250–300 g and fetuses were used in this study. Animals were kept in standardized conditions (24 °C, 12:12 h light–dark cycle, food and water ad libitum). All manipulations on animals were performed in accordance with the European Convention on the Protection of Vertebrate Animals Used for Experimental and Other Scientific Purposes (Strasburg, 1986) and approved by the Ethics Committee for Animal Research of the Koltzov Institute of Developmental Biology of RAS (approval code: Number 64 received on 22 December 2022). To obtain rats with dated pregnancies, animals were placed in time-controlled mating, and the day of conception was considered embryonic day 1 (E1). At the stage of embryo harvest, the females were sacrificed after anesthesia with 2–3% isoflurane, and embryos and placentas were eviscerated and placed in ice-cold phosphate-buffered saline (PBS). For the majority of the experiments, we utilized fetuses from 3 litters per experimental condition. Male and female fetuses were processed together.

The expression of tryptophan hydroxylase was visualized by immunohistochemistry in the intact placenta at stages E10–E20. Serotonin level was determined by High-Performance Liquid Chromatography (HPLC) in the placenta, fetal head, and trunk of untreated rats at stages E12, E14, E16, E18, E20, and E21.

Some pregnant rats were treated with LPS (25 or 250 μg/kg b.w., dissolved in saline) at stages E11, E13, E15, E17, and E19, and the placental serotonin level was measured 24 h after injection. The control rats received the same volume of saline.

Other experimental groups of animals received either LPS (250 μg/kg b.w.) or LPS (250 μg/kg b.w.) and Igs (30 min after, 6 mg per rat in saline i.v.) at stages E13 or E19 (all from Sigma-Aldrich, St. Louis, MO, USA). The placental serotonin lever was determined by HPLC 24 h after drug administration (at E14 or E20, respectively). Control rats received the same volume of saline. The Igs concentration was chosen according to previously published data [25,26,36].

### 4.2. Immunohistochemistry

Placentas were fixed in 4% paraformaldehyde in PBS at 4 °C with agitation for 4–6 h followed by rinse in PBS. After fixation samples were cryoprotected in 30% sucrose in PBS at + 4 °C with agitation for 24 h. Samples were frozen, and 15 μm cryostat sections were produced. Cryosections were preincubated in PBS containing 0.1% Triton X-100 (Sigma-Aldrich, St. Louis, MO, USA) and 1% BSA (PBSTB) (Sigma-Aldrich, St. Louis, MO, USA) for 30 min at room temperature, and then incubated overnight with primary anti-TPH antibodies diluted in PBSTB (dilution 1:1000) in a humidified chamber at 4 °C. Then, sections were washed in PBSTB three times for 10 min and incubated with secondary antibodies (dilution 1:1000) and DAPI (5 μg/mL) diluted in PBST at room temperature for 120 min, washed again three times in PBST, and mounted using Mowiol (Merck, Merck KGaA, Darmstadt, Germany, #81381) mounting medium, prepared according to manufacturer’s instructions. Images were acquired using a Zeiss LSM 880 confocal microscope (Zeiss, Jena, Germany). The choice of primary antibodies was driven by our recent studies where we confirmed by Western blotting that these antibodies detect both isoforms TPH1 and TPH2 [40].

### 4.3. High-Performance Liquid Chromatography with Fluorescent Detection (HPLC-FLD)

Tissue samples were quickly dissected on ice, weighted, homogenized with an ultrasonic homogenizer (BandelinSonopuls, Burladingen, Germany) at 4 °C in 0.1 M HClO_4_ and centrifuged at 10,000× *g* for 20 min at 4 °C. The supernatants were collected and stored at −80 °C prior to measurements of monoamine content. An Agilent 1260 Infinity II HPLC system (Agilent Technologies Inc., Waldbronn, Germany) equipped with a fluorescence detector (FLD) was used for the monoamines analysis. Analytes were separated using a reverse-phase InfinityLab Poroshell 120 EC-C18 100 mm × 4.6 mm column with a 2.7 μm particle size (Agilent Technologies Inc., Waldbronn, Germany). The column was thermostated at 30 °C. The mobile phase consisted of 0.1 M citrate–phosphate buffer, 0.25 mM 1-octanesulfonic acid sodium salt, 0.1 M EDTA, and 7% acetonitrile (pH = 2.58) (all reagents purchased from Sigma-Aldrich, St. Louis, MO, USA). The mobile phase flow rate was 1 mL/min. FLD detection was carried out at the excitation wavelength of 285 nm. The emission wavelength was set at 310 nm. Peaks of monoamines were identified by the retention time relative to the standard solutions. Serotonin was quantified by comparison of peak areas in the analyte and standard.

### 4.4. Statistical Analysis

The results are expressed as mean ± standard error of the mean (SEM) from at least three independent experiments. Statistical analysis was performed using SigmaPlot 12.0 software. The Mann–Whitney U-Test was used to compare independent groups. The difference was considered to be statistically significant at *p* < 0.05.

### 4.5. Chemicals and Reagents

Serotonin hydrochloride (Sigma Aldrich St. Louis, MO, USA #H9523), LPS (Sigma Aldrich St. Louis, MO, USA #L2630), immunoglobulins G from rat serum (Sigma Aldrich St. Louis, MO, USA #I4131), monoclonal anti-tryptophan hydroxylase antibodies, produced in mice (Sigma Aldrich St. Louis, MO, USA #T0678), Alexa Fluor 488-conjugated AffiniPure Donkey anti-mouse IgG (Jackson Immunoresearch, West Grove, PA, USA #715-545-150), DAPI (Sigma-Aldrich, St. Louis, MO, USA #D9542).

## 5. Conclusions

Our study demonstrated that the placenta is the main source of serotonin throughout most prenatal development (E12–E20) in the feto-placental unit. Susceptibility to prenatal inflammation depends on its severity and varies considerably at different stages of development. The inflammation-induced modulation of the placental serotonin level can be prevented by the administration of Igs at both early (E14) and late (E20) developmental stages.

## Data Availability

The raw data supporting the conclusions of this article will be made available by the authors on request.

## Abbreviations

LPS: lipopolysaccharide; DAPI, 4′,6-diamidino-2-phenylindol; Igs, immunoglobulins; 5-HT, 5-hydroxytryptamine or serotonin; TPH, tryptophan hydroxylase; E, embryonic day; HPLC, High-Performance Liquid Chromatography; PBS, phosphate-buffered saline; PBSTB, PBS containing 0.1% Triton X-100 and 1% BSA.
